# Contribution of the SOS response and the DNA repair systems to norfloxacin induced mutations in *E. coli*

**DOI:** 10.1007/s42995-023-00185-y

**Published:** 2023-09-21

**Authors:** Tongtong Lin, Jiao Pan, Colin Gregory, Yaohai Wang, Clayton Tincher, Caitlyn Rivera, Michael Lynch, Hongan Long, Yu Zhang

**Affiliations:** 1 Institute of Evolution and Marine Biodiversity, KLMME, Ocean University of China, Qingdao, 266003 China; 2 Laboratory for Marine Biology and Biotechnology, Laoshan Laboratory, Qingdao, 266237 China; 3 Department of Biology, Indiana University, Bloomington, 47405 USA; 4 Biodesign Center for Mechanisms of Evolution, Arizona State University, Tempe, 85281 USA; 5 School of Mathematics Science, Ocean University of China, Qingdao, 266000 China

**Keywords:** Stress response, Antibiotic-induced mutagenesis, Mutation spectrum, Linear model

## Abstract

Antibiotic-resistant bacteria severely threaten human health. Besides spontaneous mutations generated by endogenous factors, the resistance might also originate from mutations induced by certain antibiotics, such as the fluoroquinolones. Such antibiotics increase the genome-wide mutation rate by introducing replication errors from the SOS response pathway or decreasing the efficiency of the DNA repair systems. However, the relative contributions of these molecular mechanisms remain unclear, hindering understanding of the generation of resistant pathogens. Here, using newly-accumulated mutations of wild-type and SOS-uninducible *Escherichia coli* strains, as well as those of the strains deficient for the mismatch repair (MMR) and the oxidative damage repair pathways, we find that the SOS response is the major mutagenesis contributor in mutation elevation, responsible for ~ 30–50% of the total base-pair substitution (BPS) mutation-rate elevation upon treatment with sublethal levels of norfloxacin (0 ~ 50 ng/mL). We further estimate the significance of the effects on other mutational features of these mechanisms (i.e., transversions, structural variations, and mutation spectrum) in *E. coli* using linear models. The SOS response plays a positive role in all three mutational features (mutation rates of BPSs, transversions, structural variations) and affects the mutational spectrum. The repair systems significantly reduce the BPS mutation rate and the transversion rate, regardless of whether antibiotics are present, while significantly increasing the structural variation rate in *E. coli*. Our results quantitatively disentangle the contributions of the SOS response and DNA repair systems in antibiotic-induced mutagenesis.

**Supplementary Information:**

The online version contains supplementary material available at 10.1007/s42995-023-00185-y.

## Introduction

Antibiotics are one of the most important human inventions associated with disease treatment, but abuse/misuse results in widespread antimicrobial resistance in pathogenic bacteria both in the community and hospital settings, especially the emergence of multidrug-resistant strains, posing a major threat to human health (Aarestrup 2005; Ferber 2000; Rice 2009; Woodford and Ellington 2007). Mutation is a primary source of genetic variation that is used to power evolution (Pan et al. 2021). Previous studies primarily focused on resistance development and bacterial evolution under high-dose antibiotics, such as *E. coli*, *Staphylococcus aureus*, or ESKAPE pathogens treated daily with a high concentration of antibiotics (Khare and Tavazoie 2020; Mechler et al. 2015; Michiels et al. 2016). These investigations have revealed a rapid increase in the fraction of tolerant cells within the population. More recent studies have highlighted the significance of sublethal levels of antibiotics, which are commonly encountered in production and everyday life, in selecting and inducing resistance. However, the specific mechanisms of mutagenesis underlying this phenomenon remain to be fully understood (Aarestrup 2005; Andersson and Hughes 2014; Cabello 2006; Gullberg et al. 2011; Kohanski et al. 2010; Wistrand-Yuen et al. 2018; Witte 1998; Zhang et al. 2023). A recent study suggests that resistant mutants arising under sublethal antibiotic stress exhibit smaller fitness defects compared to those emerging at high doses, due to stronger competition with susceptible cells (Westhoff et al. 2017).

**Fig. 1:**
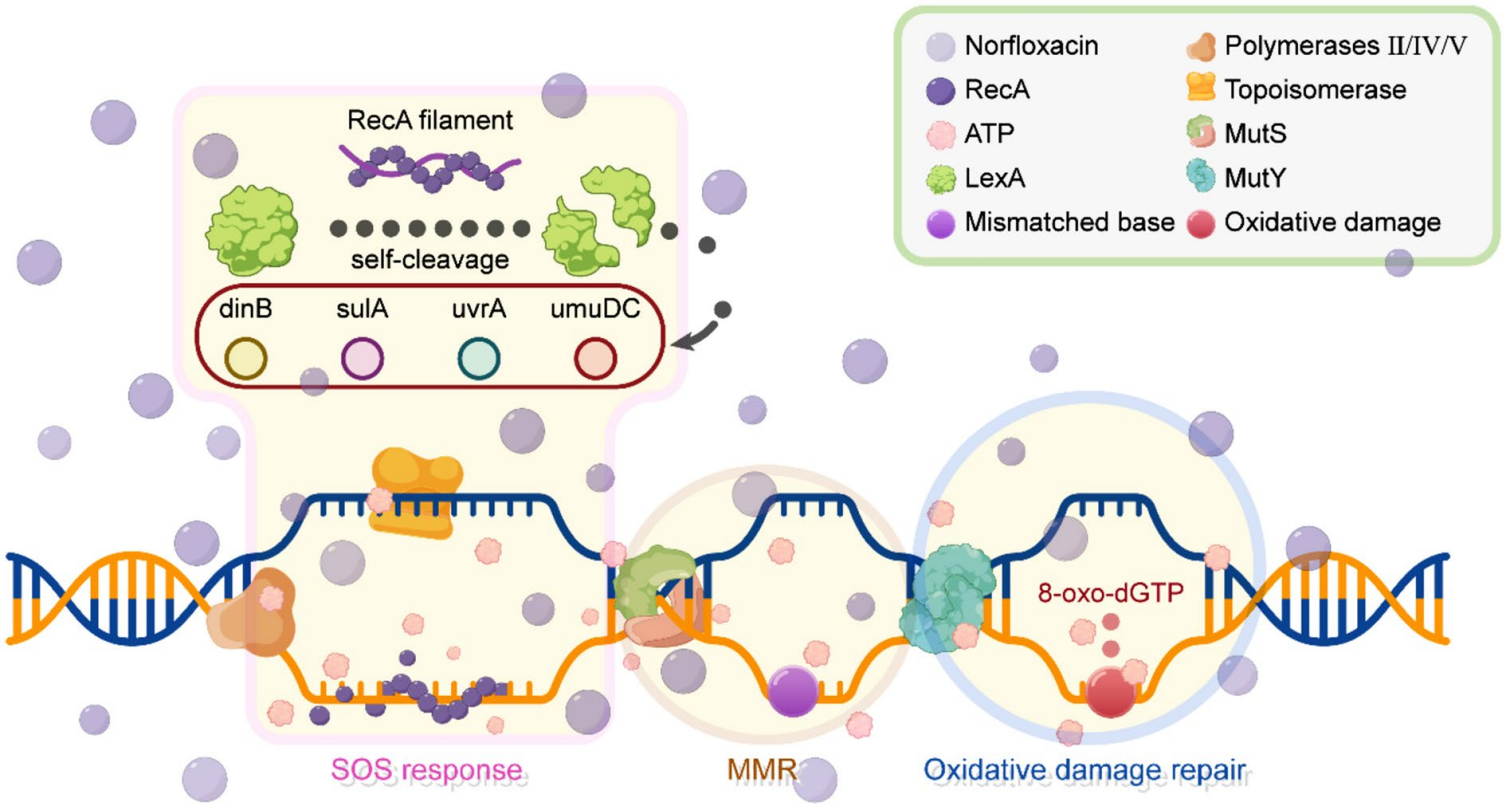
The SOS response, MMR (mismatch repair), and oxidative damage repair in bacteria. When DNA is damaged, RecA binds to single-stranded DNA (ssDNA), forming RecA filament, which induces the SOS response. The RecA filament inactivates LexA by inducing the latter to self-cleave, hence turning on the transcription of the SOS genes; MMR is a process that corrects mismatched bases in DNA strands; oxidative damage repair is a process that repairs base modifications from oxidative damage

Norfloxacin is one of the most commonly used fluoroquinolone antibacterial agents in clinics. After being taken by patients it can stay in body fluids for several days, creating a low-concentration antibiotic environment (Swanson et al. 1983). Norfloxacin affects bacterial DNA replication by inhibiting the subunit of the essential enzyme DNA gyrase, which maintains the supercoiling of DNA. This results in crosslinked protein–DNA complexes containing broken DNA, which then induces the SOS response (Cozzarelli 1980; Crumplin et al. 1984; Holmes et al. 1985; Phillips et al. 1987; Radman 1975; Wolfson and Hooper 1985). The SOS response is an anti-stress strategy in bacteria that is also responsible for stress-induced mutagenesis resulting from the use of multiple low-fidelity DNA polymerases during the response (Michel 2005; Radman 1975). About 40 genes are involved in the SOS pathway and are all repressed by the LexA protein (Fernández de Henestrosa et al. 2000). RecA (i.e., *E. coli* recombinase) forms a nucleoprotein complex (the RecA filament) that induces the response by interacting with single-stranded DNA resulting from DNA damage. The RecA filament inactivates LexA by inducing the latter to self-cleave, thereby turning on the transcription of the SOS genes (Fig. 1) (Jaszczur et al. 2016). Pribis et al. (2019) showed the effects of SOS response in fluoroquinolone mutagenesis but did not quantify the effects.

Previously, to explore antibiotic-induced mutagenesis, we ran *E. coli* K-12 MG1655 mutation-accumulation lines treated with sublethal concentrations of norfloxacin (Long et al. 2016). The results showed that the mutation rates correlate linearly with norfloxacin dose, as do the expression levels of low-fidelity DNA polymerases in the SOS response pathway, such as *polB*, *dinB*, and *umuCD*. These DNA polymerases elevate the genomic mutation rate by introducing more replication errors than pol III, the main DNA polymerase. Therefore, we hypothesize that the SOS response plays a significant role in increasing bacterial mutation rates. Our aim is to further quantify its contribution to better understand its impact.

Many other molecular mechanisms influence the mutation rate (Li et al. 2022), e.g., the heat-shock protein stress response, nucleotide-pool unbalancing, antibiotic-stimulated reactive oxygen species (ROS) (Dwyer et al. 2009). Nonetheless, in addition to the SOS response, it is primarily the DNA repair systems that have the greatest influence on bacterial mutation rates (Long et al. 2016; Oliver et al. 2002; Radman et al. 2000). However, the contributions of DNA repair systems to mutation rate elevation, especially upon antibiotic treatment, are still not quantified. DNA repair systems, such as the DNA mismatch repair (MMR) and the oxidative damage repair pathways, are also affected by the norfloxacin treatment (Fig. 1), i.e., efficiencies of MMR and oxidative damage repair decrease at higher norfloxacin concentrations (Long et al. 2016). Quantifying the respective contribution to mutation-rate elevation by these mechanisms upon antibiotic treatment is critical in understanding antibiotic-induced mutations and guiding antibiotic therapy.

In this study, we quantified the relative contribution of three mutagenesis mechanisms (i.e., the SOS response, the DNA mismatch repair, the oxidative damage repair systems) to mutation-rate elevation upon norfloxacin treatment. To do so, we applied mutation-accumulation (MA) experiments on the model bacterium *E. coli* K-12 MG1655 with different genetic backgrounds (wild-type, SOS-uninducible) (Zhao et al. 2021). In the MA experiments, dozens of parallel lines were repeatedly bottlenecked by single-colony transfers for thousands of generations, so that drift dominates selection and most mutations, even largely deleterious ones, could be accumulated in an effectively neutral fashion prior to MA-line sequencing. We also integrated published mutation datasets of the DNA-repair-deficient strains treated with the same gradients of norfloxacin (0–50 ng/mL) (Long et al. 2016). Finally, in our statistical model, we consider the potential influence of batch effects and make specific assumptions.

## Results

**Fig. 2:**
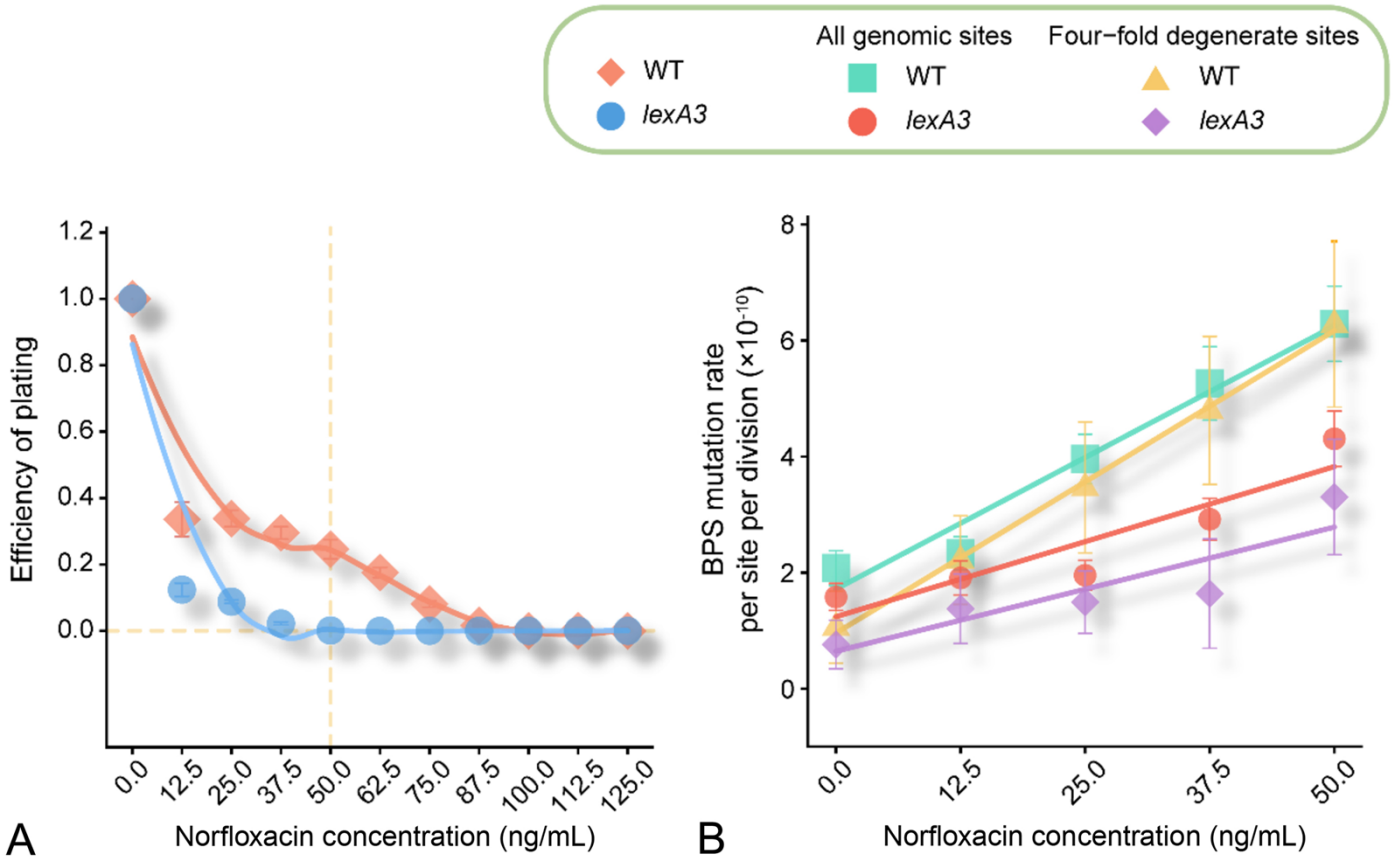
Differences in survival and mutation rates upon norfloxacin treatment, between the wild-type and the SOS-uninducible strains. **A** Efficiency of plating (EOP) of strains under different norfloxacin concentrations. The plotted lines are logistic regressions. **B** Base-pair substitution mutation rates of wild-type lines and SOS-uninducible lines treated with different doses of norfloxacin at all genomic sites and four-fold degenerate sites. Error bars are standard errors of the mean; WT, wild-type; *lexA3*, SOS-uninducible

To quantify the contribution of the SOS response to mutation-rate elevation during norfloxacin treatment, we performed MA experiments using a wild-type strain PFM2, a subculture of *E. coli* K-12 MG1655, and an SOS-uninducible strain PFM199, constructed from PFM2. For each progenitor strain, we first evaluated their survival rate over a gradient of sublethal norfloxacin concentrations, showing that the existence of the SOS response in the wild-type strain is indeed beneficial to cell survival (Fig. 2A). We also initiated five groups of MA lines from a single colony and grew 24 replicate lines per group on LB plates with 0, 12.5, 25, 37.5 and 50 ng/mL norfloxacin (Table 1). For each MA line, we performed 109 single-colony transfers on average, and after whole-genome sequencing lines with low depth of coverage (< 20 ×) or possible cross-contamination were excluded. We eventually detected 53, 64, 100, 118 and 148 base-pair substitutions (BPSs) in the five wild-type groups, and 42, 56, 53, 60 and 79 BPSs in the five SOS-uninducible groups (Table 1; Supplementary Tables S1–S4).

**Table 1:** MA-line information of *Escherichia coli* K-12 MG1655

Strain	Group	NC	Lines	Depth	Divisions	BPSs	Ins	Del	ts/tv	In/Del	Data sources
Wild-type	WA	0	19	119	2906	53	1	5	0.71	0.20	This study
						**4**	**0**	**2**	**tv = 0**	**0.00**	
Wild-type	WB	12.5	21	131	2831	64	3	4	1.29	0.75	This study
						**9**	**0**	**1**	**0.80**	**0.00**	
Wild-type	WC	25	21	115	2615	100	2	14	1.17	0.14	This study
						**13**	**0**	**2**	**0.63**	**0.00**	
Wild-type	WD	37.5	20	108	2444	118	7	22	0.97	0.32	This study
						**16**	**1**	**3**	**1.00**	**0.33**	
Wild-type	WE	50	22	100	2333	148	2	25	1.00	0.08	This study
						**22**	**0**	**3**	**0.83**	**0.00**	
*lexA3*	LA	0	20	89	2884	42	0	2	1.47	0.00	This study
						**3**	**0**	**0**	**0.00**	**Del = 0**	
*lexA3*	LB	12.5	24	131	2653	56	1	10	1.00	0.10	This study
						**6**	**0**	**1**	**1.00**	**0.00**	
*lexA3*	LC	25	24	118	2451	53	4	8	1.65	0.50	This study
						**6**	**0**	**1**	**5.00**	**0.00**	
*lexA3*	LD	37.5	22	147	2029	60	2	6	1.40	0.33	This study
						**5**	**0**	**0**	**4.00**	**Del = 0**	
*lexA3*	LE	50	20	83	1995	79	1	10	1.08	0.10	This study
						**9**	**0**	**1**	**0.50**	**0.00**	
Δ*mutS*	SA	0	12	79	763	969	105	79	50.0	1.33	Long et al. (2016)
Δ*mutS*	SB	12.5	12	55	750	1095	116	58	42.8	2.00	Long et al. (2016)
Δ*mutS*	SC	25	12	75	702	1116	118	90	26.9	1.31	Long et al. (2016)
Δ*mutS*	SD	37.5	12	96	1526	2388	184	143	16.2	1.29	Long et al. (2016)
Δ*mutS*	SE	50	12	78	696	1270	139	108	24.9	1.29	Long et al. (2016)
Δ*mutY*	YA	0	15	86	2023	234	2	4	0.11	0.50	Long et al. (2016)
Δ*mutY*	YC	12.5	19	109	2008	316	4	1	0.10	4.00	Long et al. (2016)
Δ*mutY*	YD	25	43	75	756	376	4	6	0.07	0.67	Long et al. (2016)
Δ*mutY*	YE	37.5	46	104	713	367	1	8	0.09	0.13	Long et al. (2016)
Δ*mutY*	YF	50	18	68	1735	330	11	19	0.35	0.58	Long et al. (2016)

Bold numbers show data at four-fold degenerate sites of each group

NC, norfloxacin concentration; *lexA3*, the SOS-uninducible strain; Lines, number of MA lines per group; Depth, mean depth of sequencing coverage; Divisions, mean cell divisions of MA lines; BPSs, total number of base-substitution mutations; Ins, the number of insertion mutations detected across all lines in the group; Del, the number of deletion mutations detected across all lines in the group; ts/tv ratio of transition to transversion mutations; In/Del, ratio of insertion to deletion mutation

To determine whether mutations became enriched in a few genes upon norfloxacin treatment, especially those associated with antibiotic resistance, we first examined the synonymous and nonsynonymous status of each coding-region BPS (Supplementary Tables S5–S6). We then pooled the BPS mutations in the coding regions for all genes in the wild-type MA lines treated with norfloxacin (341 BPSs in 324 genes) and those in the SOS-uninducible lines (204 BPSs in 183 genes). Then, for each gene in treated lines, we calculated the Poisson probability of the number of observed mutations in the gene greater than or equal to the expected mutation rate of the gene without norfloxacin treatment. The expected mutation number of each gene was calculated as the product of the mutation rate per nucleotide site per cell division in control lines (norfloxacin concentration is 0 ng/mL; 2.08 × 10^−10^ for the wild-type; 1.58 × 10^−10^ for the SOS-uninducible), the gene length and the total number of cell divisions in all norfloxacin-treated lines. After Bonferroni correction for multiple comparisons, significant mutation-rate elevations were observed in 29 genes in wild-type lines and 37 genes in *lexA3* lines. Notably, DNA gyrase subunit A (*gyrA*) and DNA gyrase subunit B (*gyrB*) were found to be the two that exhibited the greatest response genes, which are known to be associated with norfloxacin resistance based on their functional annotation. In short, 0.65% of the wild-type and 0.83% genes of the SOS-uninducible lines possibly experienced selection for resistance (Supplementary Tables S7–S8). Thus, even with the single-cell bottlenecking of MA experiments, antibiotics could still select for mutations in a few genes, though possibly not biasing the true mutation spectrum. To be cautious, we parsed out mutations at four-fold degenerate sites of coding regions, where any mutation would not alter the amino acid coded and hence are rarely under strong selection, and evaluated mutational patterns at these sites vs. those at all genomic sites.

### Mutational features of the wild-type vs. the SOS-uninducible lines with or without norfloxacin treatments

The SOS response is a regulatory network induced by DNA damage or DNA-replication interference and controlled by a complex circuitry involving the RecA and LexA proteins. It allows bacteria to survive DNA damage. The base-pair substitution (BPS) mutation rates of the wild-type and the SOS-uninducible strains were 2.08 × 10^–10^ and 1.58 × 10^–10^ per site per cell division, respectively, in the absence of norfloxacin. The small-indel mutation rates of the wild-type and the SOS-uninducible strains were 2.36 × 10^–11^ and 7.53 × 10^–12^ per site per cell division (Table 2). Mutation rates of the wild-type lines are significantly higher than those of the SOS-uninducible lines, i.e., SOS response increased mutation rate by 28.20% without norfloxacin, consistent with the wild-type bacteria expressing the SOS response (self-cleavage of LexA) spontaneously during unperturbed growth (Jones and Uphoff 2021; Pennington and Rosenberg 2007; Turnbull et al. 2016). The mutation rates at four-fold degenerate sites followed a similar pattern (Table 2). Thus, the SOS response is a double-edged sword, beneficial to cell survival (Fig. 2A), but introduces more mutations to bacterial genomes simultaneously.

**Table 2:** Mutation rates of the wild-type, *lexA3*, Δ*mutS* and Δ*mutY* MA lines

Strain	Group	BPSs MR	Confidence intervals		Indels MR	Confidence intervals	
Wild-type	WA	2.08	1.56	2.72	2.36	0.87	5.13
		**1.06**	**0.29**	**2.72**	**5.31**	**0.64**	**19.2**
Wild-type	WB	2.33	1.8	2.98	2.55	1.03	5.26
		**2.22**	**1.01**	**4.21**	**2.46**	**0.06**	**13.7**
Wild-type	WC	3.96	3.22	4.82	6.34	3.62	10.3
		**3.47**	**1.85**	**5.93**	**5.34**	**0.65**	**19.3**
Wild-type	WD	5.26	4.35	6.30	12.9	8.65	18.6
		**4.80**	**2.74**	**7.79**	**12.0**	**3.27**	**30.7**
Wild-type	WE	6.29	5.31	7.39	11.5	7.56	16.7
		**6.28**	**3.94**	**9.51**	**8.56**	**1.77**	**25.0**
*lexA3*	LA	1.58	1.14	2.14	0.75	0.09	2.72
		**0.762**	**0.157**	**2.23**	**0**	**0**	**9.37**
*lexA3*	LB	1.91	1.44	2.48	3.75	1.87	6.71
		**1.38**	**0.507**	**3.01**	**2.30**	**0.06**	**12.8**
*lexA3*	LC	1.96	1.47	2.56	4.43	2.29	7.74
		**1.49**	**0.548**	**3.25**	**2.49**	**0.06**	**13.9**
*lexA3*	LD	2.92	2.23	3.76	3.90	1.68	7.67
		**1.64**	**0.533**	**3.83**	**0**	**0**	**12.1**
*lexA3*	LE	4.31	3.41	5.37	6.00	2.99	10.7
		**3.31**	**1.51**	**6.27**	**3.67**	**0.09**	**20.5**
Δ*mutS*	SA	232	218	247	440	379	509
Δ*mutS*	SB	268	252	284	426	365	494
Δ*mutS*	SC	290	274	308	541	470	620
Δ*mutS*	SD	287	275	299	393	351	438
Δ*mutS*	SE	334	316	353	649	571	735
Δ*mutY*	YA	16.9	14.8	19.2	4.34	1.59	9.45
Δ*mutY*	YC	18.1	16.2	20.3	2.87	0.93	6.70
Δ*mutY*	YD	25.4	22.9	28.1	6.75	3.24	12.4
Δ*mutY*	YE	24.5	22.1	27.2	6.02	2.75	11.4
Δ*mutY*	YF	23.2	20.8	25.9	21.1	14.3	30.2

Mutation rates are in units of per nucleotide site per cell division. Bolded areas show information at four-fold degenerate sites. BPSs MR, base-substitution mutation rate (× 10^–10^); Indels MR, small indels mutation rate (× 10^–11^); confidence intervals are 95% Poisson confidence intervals (applying to tables below too) for BPSs MR (× 10^–10^) and Indels MR (× 10^–11^) respectively; group is the abbreviation for each group label of MA lines; *lexA3*, the SOS-uninducible strain. Data for the wild-type strain and *lexA3* strains are from this study; those of the Δ*mutS* and the Δ*mutY* strains are from our previous study (Long et al. 2016)

When the wild-type and the SOS-uninducible MA lines were treated with norfloxacin, BPS mutation rates at the whole-genome level showed a strong linear correlation with norfloxacin concentration (Pearson’s correlation test, *r* = 0.98, *P* = 0.0024 for the wild-type strain;* r* = 0.92, *P* = 0.03 for the SOS-uninducible strain; Tables 1 and 2; Fig. 2B; Supplementary Tables S1–S2). We then examined mutation rates at the four-fold degenerate sites of the two strains, where selection was minimal, and thus did not bias the mutation rate/spectrum. BPS mutation rates at four-fold degenerate sites of the two strains also exhibited a strong linear correlation with norfloxacin concentrations (Pearson’s correlation test, *r* = 1.00, *P* = 4.70 × 10^–5^ for the wild-type strain;* r* = 0.89, *P* = 0.04 for the SOS-uninducible strain; Tables 1 and 2; Fig. 2B; Supplementary Tables S3–S4). For either all genomic sites or four-fold degenerate sites, BPS mutation rates in the wild-type strain were significantly higher than those of the SOS-uninducible strain (paired *t*-test, *P* = 0.02 for all genomic sites; *P* = 0.03 for four-fold degenerate sites; Tables 1 and 2; Fig. 2B; Supplementary Tables S1–S4), demonstrating the contribution of the background level of SOS expression to the increase of BPS mutation rates. The small-indel mutation rate also showed a linear correlation with norfloxacin concentrations in both the wild-type and the SOS-uninducible strains (Pearson’s correlation test, *r* = 0.92, *P* = 0.03 for the wild-type strain;* r* = 0.88, *P* < 0.05 for the SOS-uninducible strain; Tables 1 and 2; Supplementary Fig. S1; Tables S1–S2).

**Fig. 3:**
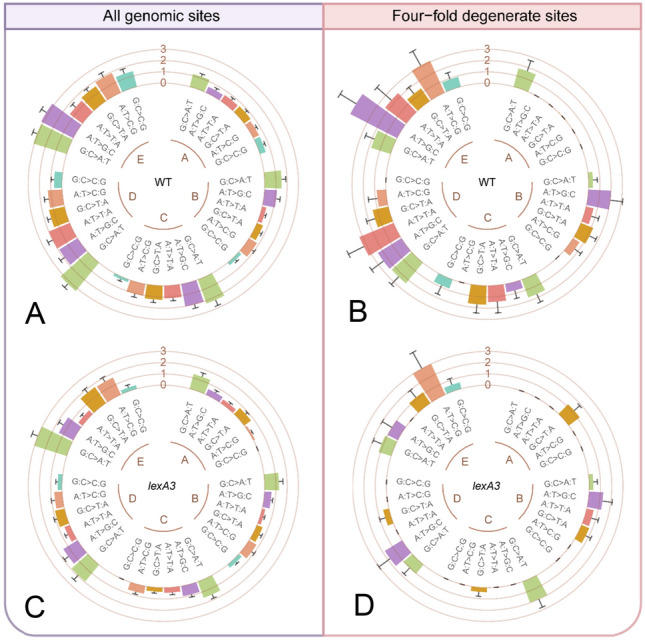
Mutation spectra of the wild-type and the SOS-uninducible lines treated with different doses at all genomic sites and four-fold degenerate sites. Error bars denote SEM. A–E in each figure represent norfloxacin concentrations of 0, 12.5, 25, 37.5, 50 ng/mL respectively. The tracks show base-pair substitution mutation rates per site per cell division (× 10^–10^). **A** Mutation spectra of wild-type lines at all genomic sites. **B** Mutation spectra of wild-type lines at four-fold degenerate sites. **C** Mutation spectra of SOS-uninducible lines at all genomic sites. **D** Mutation spectra of SOS-uninducible lines at four-fold degenerate sites. WT, wild-type; *lexA3*, SOS-uninducible

The mutation spectrum is a major determinant in genome-architecture evolution (Long et al. 2018b). To understand bacterial genome evolution under antibiotic stress, it is essential to evaluate norfloxacin’s effects on the mutation spectrum. We first analyzed the mutation spectra of the wild-type and the SOS-uninducible MA lines without norfloxacin stress (WA and LA) for all genomic sites and four-fold degenerate sites (Fig. 3; Supplementary Fig. S2; Tables S1–S4). The G:C$\to$A:T transitions were the most abundant mutation-type in both sets of MA lines, with the main difference between the two strains being A:T$\to$C:G and G:C$\to$C:G transversions. However, at four-fold degenerate sites, due to the small number of mutations accumulated, G:C$\to$A:T transitions and G:C$\to$T:A transversions were the only non-zero mutations in the wild-type lines and in the SOS-uninducible lines, respectively (Fig. 3; Supplementary Fig. S2). The transition/transversion (ts/tv) and the insertion/deletion (In/Del) ratios are all shown in Table 1. There was no significant correlation between the ts/tv ratio and the norfloxacin concentration in the wild-type lines. When all genomic sites were considered, the highest ts/tv ratio of the wild-type control and the treatments was 1.29 (WB group) but decreased to 1.00 (WD group) at four-fold degenerate sites. For the SOS-uninducible MA line groups, all ts/tv ratios were above 1.00 and the highest was 1.65 (LC group) for all genomic sites. While at four-fold degenerate sites, the lowest ts/tv ratio was 0.00 in group LA, and the maximum reached 5.00 in group LC. The large fluctuation may be due to the small mutation number.

### Quantifying the contribution of the SOS response, DNA mismatch repair (MMR) and oxidative damage repair to mutation-rate elevation

**Fig. 4:**
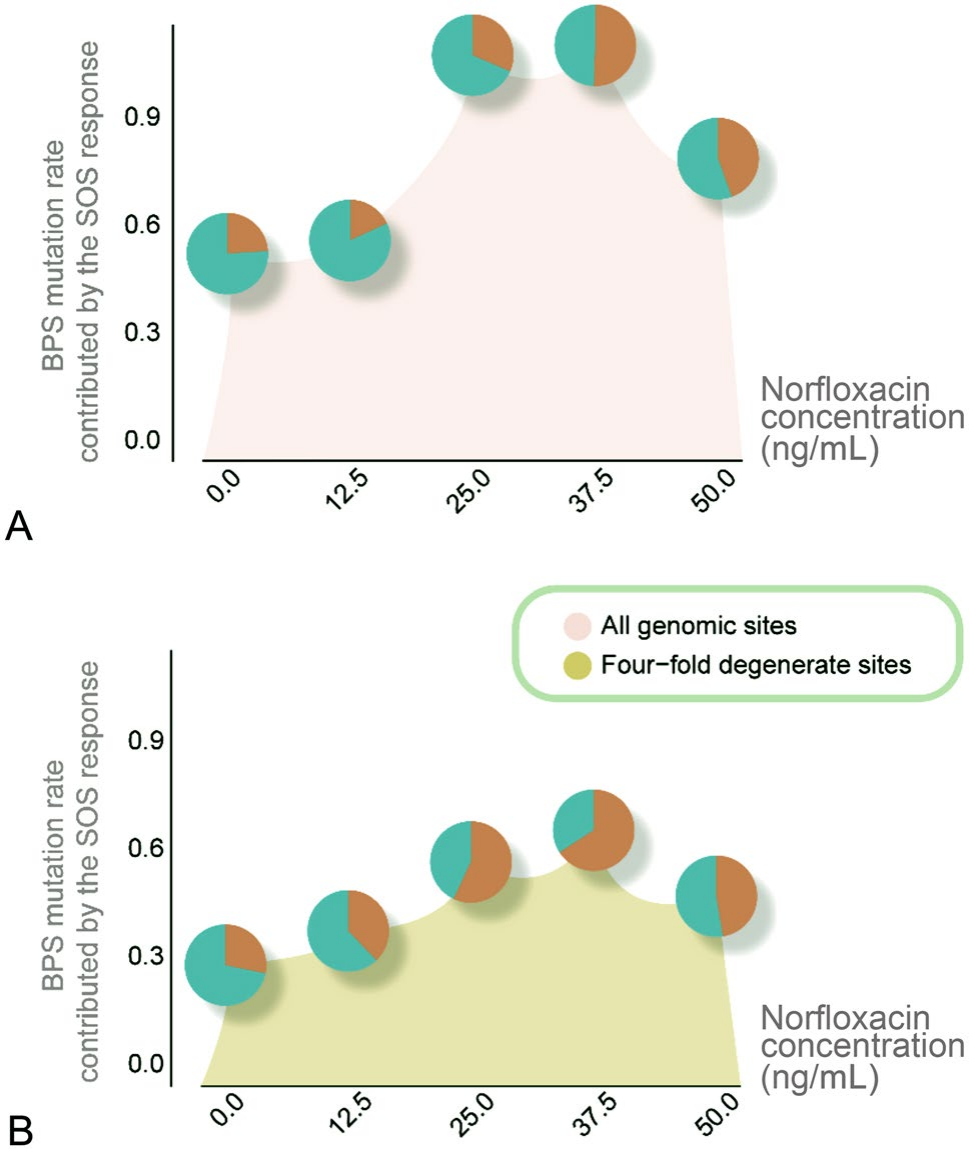
Proportion of BPS mutation rate contributed by the SOS response under different norfloxacin doses at all genomic sites and four-fold degenerate sites. The red part in the pie chart is the contribution proportion of the SOS response. **A** Proportion of BPS mutation rate from the SOS response at all genomic sites, when treated with different doses of norfloxacin. **B** The proportion of BPS mutation rate the SOS response contributed at four-fold degenerate sites at different doses of norfloxacin

At each norfloxacin concentration, the difference in mutation rate between the wild-type and the SOS-uninducible lines directly reveals the mutation rate elevated by the SOS response. The BPS mutation rate was increased by norfloxacin treatment. However, the degree of this elevation did not show a significant linear correlation with the doses (*r* = 0.70, *P* = 0.18 for four-fold degenerate sites;* r* = 0.48, *P* = 0.41 for all genomic sites; Fig. 4). However, there was a non-linear response with concentration, reaching a peak of ~ 65% when exposed to 37.5 ng/mL at four-fold degenerate sites (maximally 50% at 25 ng/mL at all genomic sites), declining to 47% at 50 ng/mL, and reaching the lowest values at 0 and 12.5 ng/mL (Supplementary Tables S9–S10). Moreover, ts/tv and In/Del ratios also peaked at their maximum levels at 37.5 ng/mL. We thus speculate that 37.5 ng/mL norfloxacin could be a critical cutoff concentration for the overall effects of the complex mutagenesis mechanisms. We also calculated the change of mutation spectrum and small-indel mutation rate caused by the SOS response at all genomic sites and four-fold degenerate sites (Supplementary Figs. S3–S4; Tables S9–S11). We can then conclude that the SOS response does contribute to the mutation rate elevation and mutation spectrum change in *E. coli*.

**Fig. 5:**
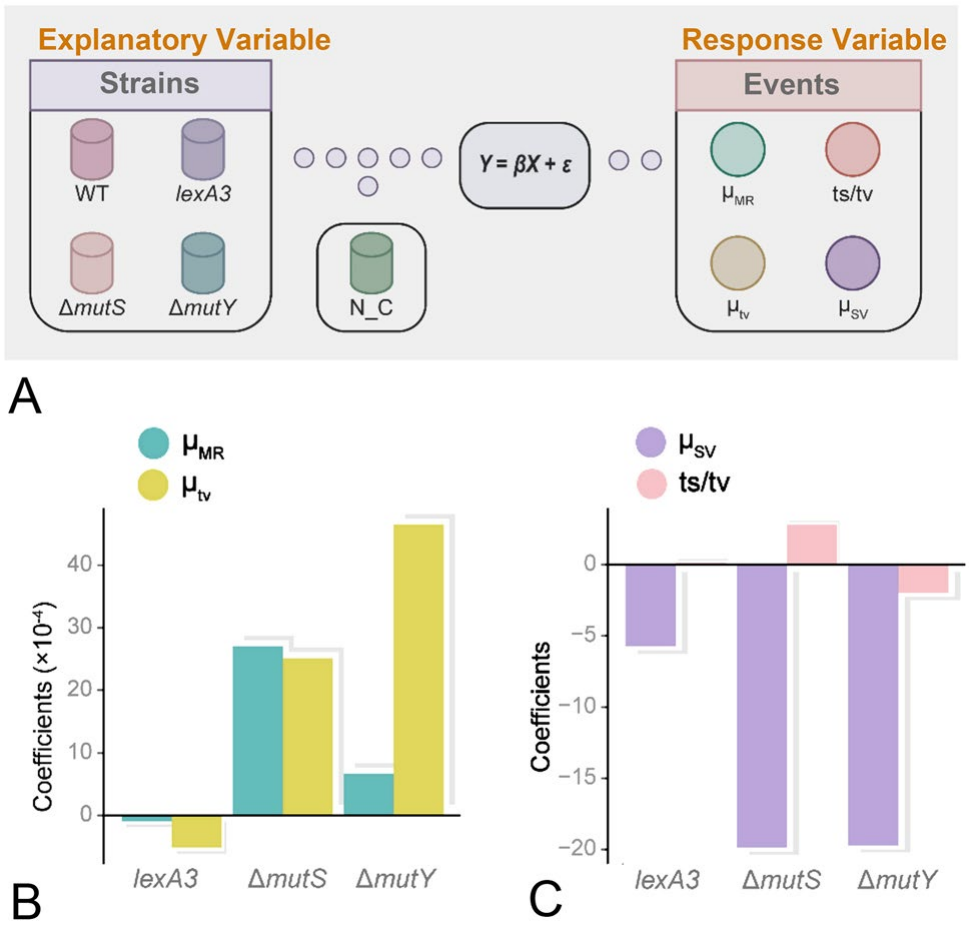
The modeling approach and the estimated relationships by the statistical models. **A** The modeling approach to our linear regression model predicts the probability of four strains from a range of features. Model 1 only uses strain as the explanatory variable, and Model 2 uses both strain and N_C (norfloxacin concentration) as explanatory variables. The response variables, μ_MR_ is BPS mutation rate, μ_tv_ is transversion rate, ts/tv is transition/transversion ratio, and μ_SV_ is structural variation rate. **B** Predictive coefficients of three strains on BPS mutation rate (μ_MR_) and transversion rate (μ_tv_) from the linear Model 1. **C** Predictive coefficients of three strains on transition/transversion ratio (ts/tv) and structural variation rate (μ_SV_) from the linear Model 1

In addition to the stress response, other critical molecular mechanisms might be involved, such as MMR and the oxidative damage repair systems, the absence of which is known to cause significant changes in mutation rates and their repair efficiency could also be compromised by norfloxacin (Long et al. 2016). The relative contribution of the three mechanisms (SOS response, MMR, the oxidative damage repair) in elevating mutation rate without considering the impact of antibiotics is not yet quantified. For that purpose, assuming no interaction between any mechanism, we first used mutation datasets of the wild-type and the SOS-uninducible MA lines generated in this study, and those of the wild-type, MMR-deficient (Δ*mutS*) and the adenine DNA glycosylase–deficient (Δ*mutY*) MA lines from our previous study (Long et al. 2016), to fit a linear statistical model. The fitted model for the response variable (mutation rate) is (Fig. 5A; see details in Model 1, Statistical Model, Materials and Methods section):

$${{\mu }_{MR}}^{*}=-2.605-8.867\times {10}^{-5}lexA3+2.698{\times 10}^{-3}\Delta mutS+6.620{\times 10}^{-4}\Delta mutY.$$

Here, *lexA3,* Δ*mutS* and Δ*mutY* are all dummy variables that only take the value 0 or 1. When *lexA3*, Δ*mutS* or Δ*mutY* equals 1, the strain is the SOS-uninducible strain, the MMR-deficient strain, or the adenine DNA glycosylase–deficient strain, respectively. To satisfy the assumptions of the linear model, a Box-Cox Transformation was applied to the BPS mutation rates (MR) when we fit the model. ${{\mu }_{MR}}^{*}$ represents the value of MR after the Box-Cox Transformation with $\lambda =0.384$. Consequently, when *lexA3*, Δ*mutS* and Δ*mutY* are all equal to 0, ${{\mu }_{MR}}^{*}$ represents the fitted mutation rate of the wild-type strain after the transformation.

The coefficients of the fitted model, *P* and 95% confidence intervals for the coefficients are shown in Table 3. The positive coefficients for Δ*mutS* and Δ*mutY* demonstrate that Δ*mutS* and Δ*mutY* strains had positive effects on mutation rates in *E. coli* (*P* < 2 × 10^–16^; Tables 3 and 4; Fig. 5B). However, the SOS-uninducible genotype (*lexA3*) played an opposite role in the mutation rate with a negative coefficient (*P* = 2.83 × 10^–4^; Tables 3 and 4; Fig. 5B). The 95% confidence intervals for coefficients of *lexA3*, Δ*mutS* and Δ*mutY* strains also supported the results above (Table 3). Consequently, consistent with their biological functions, the MMR and the oxidative damage repair systems (when active) decrease the mutation rate. The MMR has a more substantial effect than the oxidative damage repair system (Fig. 5B). On the contrary, active SOS elevates the mutation rate as expected (Long et al. 2016).

**Table 3:** The information of coefficients for the fitted Model 1 with the response variable being BPS mutation rate

Strain	Coefficients	*P*	Confidence intervals	
*lexA3*	− 8.87 × 10^–5^	2.83 × 10^–4^***	− 1.36 × 10^–4^	− 0.41 × 10^–4^
Δ*mutY*	6.62 × 10^–4^	< 2.00 × 10^–16^ ***	6.18 × 10^–4^	7.06 × 10^–4^
Δ*mutS*	2.70 × 10^–3^	< 2.00 × 10^–16^ ***	2.64 × 10^–3^	2.76 × 10^–3^

The response variable for the fitted model is the mutation rate with Box-Cox Transformation ($\lambda =0.384$), and the explanatory variable is molecular mechanism. The coefficients represent the difference in mutation rate between wild-type and each mutant strain, and the *P* and confidence intervals reveal coefficients’ significance. *lexA3*, the SOS-uninducible strain

**Table 4:** The effect of each mechanism on different mutational features (Model 1)

Features	Mechanisms	Model type	Effects	Significance
μ_MR_	SOS response	1	+	Yes
	MMR	1	–	Yes
	Oxidative damage repair	1	–	Yes
ts/tv	SOS response	1	–	No
	MMR	1	–	Yes
	Oxidative damage repair	1	+	Yes
μ_tv_	SOS response	1	+	Yes
	MMR	1	–	Yes
	Oxidative damage repair	1	–	Yes
μ_SV_	SOS response	1	+	No
	MMR	1	+	Yes
	Oxidative damage repair	1	+	Yes

Features, mutational features; “ + ”, positive effects; “–”, negative effects. Significance is determined based on *P* < 0.05. μ_MR_, BPS mutation rate; μ_tv_, transversion rate; ts/tv, transition/transversion ratio; μ_SV_, structural variation rate

Furthermore, to estimate the respective contribution of the three mechanisms (SOS response, MMR, the oxidative damage repair) to mutation-rate elevation upon antibiotic treatments, the norfloxacin concentration, and the interaction of norfloxacin concentration and the mechanisms were added to the linear model (Fig. 5A; see details in Model 2, Statistical Model, Materials and Methods section). After fitting the model, we obtained:

$$\begin{aligned}{{\mu }_{MR}}^{*} &=-2.605+6.050\times {10}^{-6}N\_C-4.366{\times 10}^{-5}lexA3+2.660{\times 10}^{-3}\Delta mutS+7.274{\times 10}^{-4}\Delta mutY-1.707{\times 10}^{-6}N\_C\cdot lexA3+1.583{\times 10}^{-6}N\_C\cdot\Delta mutS-2.928{\times 10}^{-6}N\_C\cdot\Delta mutY \\ &=\left\{\begin{aligned}& -2.6050+6.050{\times 10}^{-6}N\_C \quad\text{if wild-type}\\ & -2.6049+4.343{\times 10}^{-6}N\_C \quad \text{if SOS-uninducible}\\ -2.6022+7.634{\times 10}^{-6}N\_C\quad \text{ if }\Delta mutS\\ & -2.6041+3.122{\times 10}^{-6}N\_C \quad \text{ if }\Delta mutY\end{aligned}\right.\end{aligned}$$

Here, *N_C* is the norfloxacin concentration in units of ng/mL norfloxacin (Fig. 5A). The information of coefficients for the fitted model is shown in Supplementary Table S12 and Fig. S5A. An ANOVA test was applied to determine whether Model 2 was significantly better at capturing the data than Model 1. Supplementary Fig. S5A shows the estimated relationships between norfloxacin concentration and mutation rate for different strains in Model 2 (Supplementary Table S12). It revealed that change in *N_C* had different effects on mutation rate of different strains. We noted that the slope for Δ*mutS* was higher than wild-type*,* SOS-uninducible or Δ*mutY*. This suggested that the increases in norfloxacin concentration were associated with more significant increases in mutation rate among the MMR-deficient MA lines compared to others, inferring that when DNA replication is disturbed by norfloxacin treatment, MMR is still the main limiting factor of the mutation rate. Moreover, the strength of the SOS response increasing and MMR decreasing mutation rate both became higher as the norfloxacin concentration went up. But the negative effects of the oxidative damage repair to mutation rate elevation hardly changed with the norfloxacin concentration (Table 5; Supplementary Fig. S5A).

**Table 5:** The effects of each mutagenesis mechanism on mutational features, and the trend with norfloxacin doses (Model 2)

Features	Mechanisms	Model type	Effects	Trend
μ_MR_	SOS response	2	+	↑
	MMR	2	–	↑
	Oxidative damage repair	2	–	→
ts/tv	SOS response	2	–	↑
	MMR	2	–	↑
	Oxidative damage repair	2	+	↓
μ_tv_	SOS response	2	+	↑
	MMR	2	–	↓
	Oxidative damage repair	2	–	↑
μ_SV_	SOS response	2	+	↑
	MMR	2	+	↓
	Oxidative damage repair	2	+	↑

Features, mutational features; “ + ”, positive effects; “–” negative effects. Significance is determined based on *P* < 0.05. μ_MR_, BPS mutation rate; μ_tv_, transversion rate; ts/tv, transition/transversion ratio; μ_SV_, structural variation rate. Trend, the slope difference between the mechanism vs. the wild-type, reveals the trend of mechanism’s effects vs. norfloxacin doses; “↑”, the mutational feature increases as the norfloxacin concentration goes up; “↓”, the mutational feature decreases as the norfloxacin concentration increases, “ → ”, the mutational feature does not change as the norfloxacin concentration increases

We also explored the contribution of the above molecular mechanisms to the mutation spectrum (Tables 4 and 5; Fig. 5B, C; Supplementary Table S13; Fig. S5; details are in Supplementary File). Using the transition to the transversion ratio (ts/tv) data of all MA lines of the four strains (MA lines with both ts and tv being non-zeros were chosen) (Table 4; Fig. 5C; Supplementary Table S13), we first quantified the molecular mechanisms vs. the mutation spectrum. The SOS response did not significantly change the mutation spectrum, while the MMR and the oxidative damage repair did change the mutation spectrum by increasing or decreasing the ts/tv ratio respectively (*P* < 2 × 10^–16^). After the norfloxacin concentration was considered, the SOS response and the MMR decreased the ts/tv ratio (Table 5; Supplementary Fig. S5B). In addition, the oxidative damage repair significantly increased the ts/tv ratio, but such effect weakened as the norfloxacin concentration increased. Similarly, we also explored the effects of the molecular mechanisms vs. other mutational features, such as the transversion rate and the structural variation rate. The detailed results are in Supplementary File, Tables 4 and 5, Fig. 5B and C, Supplementary Tables S15–S20 and Supplementary Fig. S5C and D.

## Discussion

Our study revealed mutational responses to sublethal concentrations of norfloxacin with or without the presence of the SOS response at all-scales (including BPS mutations, indels, deletions, and structural variations). Meanwhile, based on linear modelling with de novo mutations in 103 wild-type and 110 SOS-uninducible *E. coli* MA lines reported here, as well as those of 60 MMR-deficient and 141 oxidative-damage-repair-deficient lines obtained from published work that applied the same procedures (Long et al. 2016), we quantified the contribution of the three major mutagenesis mechanisms (i.e., the SOS response, compromised MMR and oxidative damage repair) accounting for norfloxacin-induced mutagenesis. We supported the associations between BPS mutation rates and the three mutagenesis mechanisms: MMR and the oxidative damage repair systems significantly reduced BPS mutation rates, and the effects tended to be enhanced with the increase of norfloxacin concentrations. But the SOS response increased mutation rate, regardless of being in the presence or absence of norfloxacin, and its impact on mutation-rate elevation increased as norfloxacin concentration went up. Among the three mechanisms, MMR is the most powerful biological determinant of the mutation rate, repairing more than 99% spontaneous pre-mutations in most studied bacteria (Long et al. 2018a). In the presence of fluoroquinolones, it has been reported that the failure of type II topoisomerases to combine will directly result in double-stranded breaks (Aldred et al. 2014; Wohlkonig et al. 2010), which will directly cause the response of intracellular mechanisms and may be one of the ways to form mutations. Thus, fluoroquinolones can accelerate the evolution of the treated bacteria through diverse mechanisms. Nevertheless, mechanisms associated with mutagenesis may not be limited to those we focused on in this study but also heat-shock protein stress response, nucleotide-pool unbalancing, mobile genetic elements (Bjedov et al. 2003; Foster 2007; Genther et al. 1977; Guisbert et al. 2008; Partridge et al. 2018). A comprehensive investigation including more potential mechanisms would provide a more accurate picture of antibiotic-induced mutagenesis.

Furthermore, the models in this study may require refinement because the complicated interactions between SOS response and other mechanisms were not accounted for. For example, MMR can correct the majority of SOS-induced mutations (Lewis et al. 2021), and the SOS response can also repair some oxidative DNA damages in either an error-free or error-prone manner. More knockout strains, especially those double-knockouts carrying only one active mechanism, would help resolve the complex interactions for ideal modelling. Nonetheless, the results obtained by the current models are consistent with conclusions of previous studies, supporting the reliability of the current models. In addition, the genetic background of the SOS-uninducible strain PFM199, *recA730 lexA3* Δ*sulA* also brings in complications. There is concern whether such genetic constructs with *lexA3* plus the mutant allele *recA730* and deletion of *sulA* would bring in complications, i.e., more biological functions leading to mutagenesis besides the SOS response. The lower genomic mutation rate of PFM199 than that of the wild-type without norfloxacin treatment removes such possibility (Table 2, ‘WA’ and ‘LA’). This is further supported by our findings that mutation rate is elevated by the SOS response, which is consistent with studies using other methods (Cirz et al. 2005; Mo et al. 2016).

Wijker and Lafleur (1998) proposed that transversions were elevated when UV-light induced the SOS response. Our model, which combines MA data of the SOS-uninducible and the wild-type strains, supports that the SOS response significantly increases the transversion rate during growth with or without norfloxacin treatment (Fig. 5B; Supplementary Fig. S5C; Table S15). Moreover, in the absence of external stress, MMR and oxidative damage repair systems mainly determine the mutation spectrum (quantified by the ts/tv ratio), even though their effects were counteractive, i.e., MMR increases the ts/tv ratio, while oxidative damage repair reduces it (Table 4; Fig. 5C). However, as norfloxacin concentration goes up, the positive effects for oxidative damage repair become weaker (Table 5; Supplementary Fig. S5B). Thus, it can be inferred that the SOS response is far overshadowed by the DNA oxidative repair system in determining the mutation spectrum under norfloxacin treatment.

Structural variations (SVs) are critical for studying genome evolution, such as speciation and genome reduction (Delneri et al. 2003; Raeside et al. 2014; Sloan and Moran 2013) but frequently neglected due to analytical barriers. The SV analysis results reveal that the SOS response increases the SV rate and helps bacteria survive DNA damage when there is no norfloxacin stress. When MMR and oxidative damage repair mechanisms are present, SVs are more likely to occur whether norfloxacin is present or not; this demonstrates that SVs are beyond their scope of repair. As the norfloxacin stress increases, the SOS response and the oxidative damage repair elevate SV rates, while MMR plays the opposite role.

To conclude, using statistical models and MA lines of three mutant strains and the wild-type, we studied the influence on four mutational features: BPS mutation rate, mutation spectrum, transversion rate and structural variation rate by the SOS response, MMR and oxidative damage repair. None of the mechanisms accounts for 100% of any mutational feature, demonstrating that the other mechanisms are still coordinating when one mechanism is lacking. This study tips the iceberg towards the contribution of the complex molecular mechanisms involved in antibiotic-induced mutagenesis and fills the gap between quantitative and molecular genetics of the phenomenon.

## Materials and methods

### Strains and media

The *Escherichia coli* K-12 MG1655 wild-type (PFM2) and SOS-uninducible strains (PFM199) were kindly provided by Patricia Foster’s lab, Indiana University, Bloomington. LexA3 of PFM199 (*recA730 lexA3* Δ*sulA*) cannot undergo auto-proteolysis and thus, bacteria are unable to express the SOS genes (Niccum et al. 2020). LB agar plates and stock solutions of norfloxacin were made according to the manufacturers’ instructions (Solarbio, L8290).

### The efficiency of plating (EOP) upon norfloxacin treatments

Cells were cultured for 16 h and maintained in exponential phase, after which they were serially diluted. About 1500 cells were then plated onto LB plates containing 0, 12.5, 25, 37.5, 50, 62.5, 75, 87.5, 100, 112.5 and 125 ng/mL norfloxacin, with six replicates for each concentration. Colony-forming units were then counted after 24 h cultivation at 37 °C. EOP was then calculated by dividing the colony-forming units (CFU) with those from a blank control.

### MA experiments

We followed the protocols by Kibota and Lynch (1996). Briefly, wild-type and SOS-uninducible MA lines were initiated from a single ancestral colony of PFM2 and PFM199, respectively. For each strain, five groups with 24 MA lines were treated with 12.5 ng/mL progression of norfloxacin concentrations (labeled as WA/LA: 0 ng/mL, WB/LB: 12.5 ng/mL, WC/LC: 25 ng/mL, WD/LD: 37.5 ng/mL, WE/LE: 50 ng/mL; W is for wild-type, L is for *lexA3*; Table 1). We single-colony transferred all MA lines daily for ~ 110 times per line.

About every ten transfers, we estimated the number of cell divisions that the MA lines had passed through by CFU of single diluted colonies from five randomly selected MA lines for each group. The total cell division number of each MA line is the product of the grand mean of all cell division estimates and the total transfers of each line.

### DNA extraction, library construction and genome sequencing

We extracted genomic DNA of *E. coli* MA lines using the Wizard Genomic DNA Purification Kit (Promega). DNA libraries were generated using the protocol of Li et al. (2019), but replacing the library kit with the Nextera DNA Library Preparation Kit for Illumina. HiSeq2500 PE150 sequencing with an insert size of 300 bp was then performed by the Hubbard Center for Genome Studies, University of New Hampshire (Durham, NH). After excluding cross-contaminated and depth of coverage < 20 × lines, the median depths of sequencing coverage are shown in Table 1.

### Mutation analyses and statistics

For raw reads of all final MA lines, we used Trimmomatic-0.38 to trim off adaptors and then mapped the clean reads to the reference genome using BWA-0.7.17 mem (GenBank genome accession number: NC_000913.3 for *E. coli* K-12 MG1655) (Bolger et al. 2014; Li 2013). Duplicate reads were then removed using picard-tools-2.17.2. SNPs and indels were discovered by HaplotypeCaller, using standard hard filtering parameters described by GATK-4.1.2.0 Best Practices recommendations (DePristo et al. 2011; McKenna et al. 2010). Mutation curation was done using the Integrative Genomics Viewer, IGV_2.8.12 (Thorvaldsdóttir et al. 2013).

All statistics and illustrations were done with R packages ggplot2, ggpubr, dplyr, forcats, gridExtra, viridis, and MASS in R-4.0.2 (R Development Core Team 2013).

### Statistical modelling

A linear regression model was used to estimate the significance of the effects of SOS response, MMR and DNA oxidative damage repair on the corresponding response variable. In this model, the continuous variable (denoted as $${Y}_{i})$$ is the response variable, while molecular mechanism is explanatory variable that is categorical with four levels: wild-type, *lexA3*, Δ*mutS* and Δ*mutY*. Suppose we have *n* observations, wherein $$i=\text{1,2},3,\dots ,n$$. In this situation, three dummy variables can be created. The first could be:

$$ lexA3_{i} = \left\{ {\begin{array}{*{20}l} {1\quad {\text{if}}\;i{\text{th}}\;{\text{observation}}\;{\text{is}}\;lexA3\left( {\text{SOS-uninducible}} \right)} \\ {0\quad {\text{if }}i{\text{th observation is wild-type}}.} \\ \end{array} } \right. $$

The second could be:

$$ \Delta mutS_{i} = \left\{ {\begin{array}{*{20}l} {1\quad {\text{if }}i{\text{th observation is }}\Delta mutS\left( {\text{MMR-deficient}} \right)} \\ {0\quad {\text{if }}i{\text{th observation is wild-type}}} \\ \end{array} } \right.. $$

The third could be:

$$ \Delta mutY_{i} = \left\{ {\begin{array}{*{20}l} {1\quad {\text{if }}i{\text{th observation is }}\Delta mutY({\text{adenine DNA glycosylase}}{-}{\text{deficient}})} \\ {0\quad {\text{if }}i{\text{th observation is wild-type}}} \\ \end{array} } \right. $$

The linear model can be written as Eq. (1):

1 $$\begin{aligned}{Y}_{i} & ={\beta }_{0}+{\beta }_{1}l{exA3}_{i}+{\beta }_{2}{\Delta mutS}_{i}+{\beta }_{3}{\Delta mutY}_{i}+{\epsilon }_{i}\\ &= \left \{ \begin{aligned}&{\beta }_{0}+{\epsilon }_{i}\quad \text{if }i\text{th observation is wild-type}\\ & {\beta }_{0}+{\beta }_{1}+{\epsilon }_{i}\quad \text{if }i\text{th observation is }lexA3\\ & {\beta }_{0}+{\beta }_{2}+{\epsilon }_{i}\quad \text{if }i\text{th observation is }\Delta mutS\\ & {\beta }_{0}+{\beta }_{3}++{\epsilon }_{i}\quad \text{if }i\text{th observation is }\Delta mutY \end{aligned}\right. \end{aligned}$$

Here, ${\beta }_{0}$ can be interpreted as the value of the response variable among the observations of wild-type, ${\beta }_{1}$ as the difference in the value of the response variable between the observations of wild-type and SOS-uninducible, ${\beta }_{2}$ as the difference in the value of the response variable between the observations of wild-type and Δ*mutS*, and ${\beta }_{3}$ as the difference in the value of the response variable between the observations of wild-type and Δ*mutY*.

To estimate the effects of SOS response, Δ*mutS* and Δ*mutY* on the value of the response variable under different norfloxacin concentrations. The norfloxacin concentration, denoted by *N_C*, and the interaction of norfloxacin concentration and molecular mechanism are added to the model. Here, *N_C* is a quantitative variable and molecular mechanism is a qualitative variable. Then the linear model can be written as Eq. (2). The regression lines have different intercepts (i.e., ${\beta }_{0}$ vs. ${\beta }_{0}+{\beta }_{2}$) and different slopes (i.e., ${\beta }_{1}$ vs. ${\beta }_{1}+{\beta }_{5}$).

2 $$\begin{aligned}{Y}_{i} & ={\beta }_{0}+{\beta }_{1}{N\_C}_{i}+{\beta }_{2}{lexA3}_{i}+{\beta }_{3}{\Delta mutS}_{i}+{\beta }_{4}{\Delta mutY}_{i}+{\beta }_{5}{N\_C}_{i}\cdot {lexA3}_{i}\\ & \quad +{\beta }_{6}{N\_C}_{i}\cdot {\Delta mutS}_{i}+{\beta }_{7}{N\_C}_{i}\cdot {\Delta mutY}_{i}+{\epsilon }_{i}\\ &=\left \{\begin{aligned}&{\beta }_{0}+{\beta }_{1}{N\_C}_{i}+{\epsilon }_{i} \quad \text{if }i\text{th observation is wild-type}\\ & {\beta }_{0}+{\beta }_{2}+{({\beta }_{1}+{\beta }_{5})}{N\_C}_{i}+{\epsilon }_{i} \quad \text{if }i\text{th observation is }lexA3\\ & {\beta }_{0}+{\beta }_{3}+{({\beta }_{1}+{\beta }_{6})}{N\_C}_{i}+{\epsilon }_{i}\quad \text{if }i\text{th observation is }\Delta mutS \\ & {\beta }_{0}+{\beta }_{4}+{({\beta }_{1}+{\beta }_{7})}{N\_C}_{i}+{\epsilon }_{i}\quad \text{if }i\text{th observation is }\Delta mutY \end{aligned} \right. \end{aligned}$$

### Supplementary Information

Below is the link to the electronic supplementary material.

Supplementary file1 (DOCX 1245 KB)

Supplementary file2 (XLSX 206 KB)

Supplementary file3 (DOCX 44 KB)

## Data Availability

All MA line raw genome sequences are deposited in NCBI SRA with BioProject No. PRJNA301160, and Study No. SRP066119.
