# Supplementary material for: Contribution of the SOS response and the DNA repair systems to norfloxacin induced mutations in E. coli
Source: Mar Life Sci Technol. 2023 Sep 21;5(4):538–50. doi: 10.1007/s42995-023-00185-y (PMC10689325; doi:10.1007/s42995-023-00185-y)
Supplement: Supplementary file 1 — Supplementary file1 (DOCX 1245 KB) [file 42995_2023_185_MOESM1_ESM.docx]

**Supplementary Figures**

**
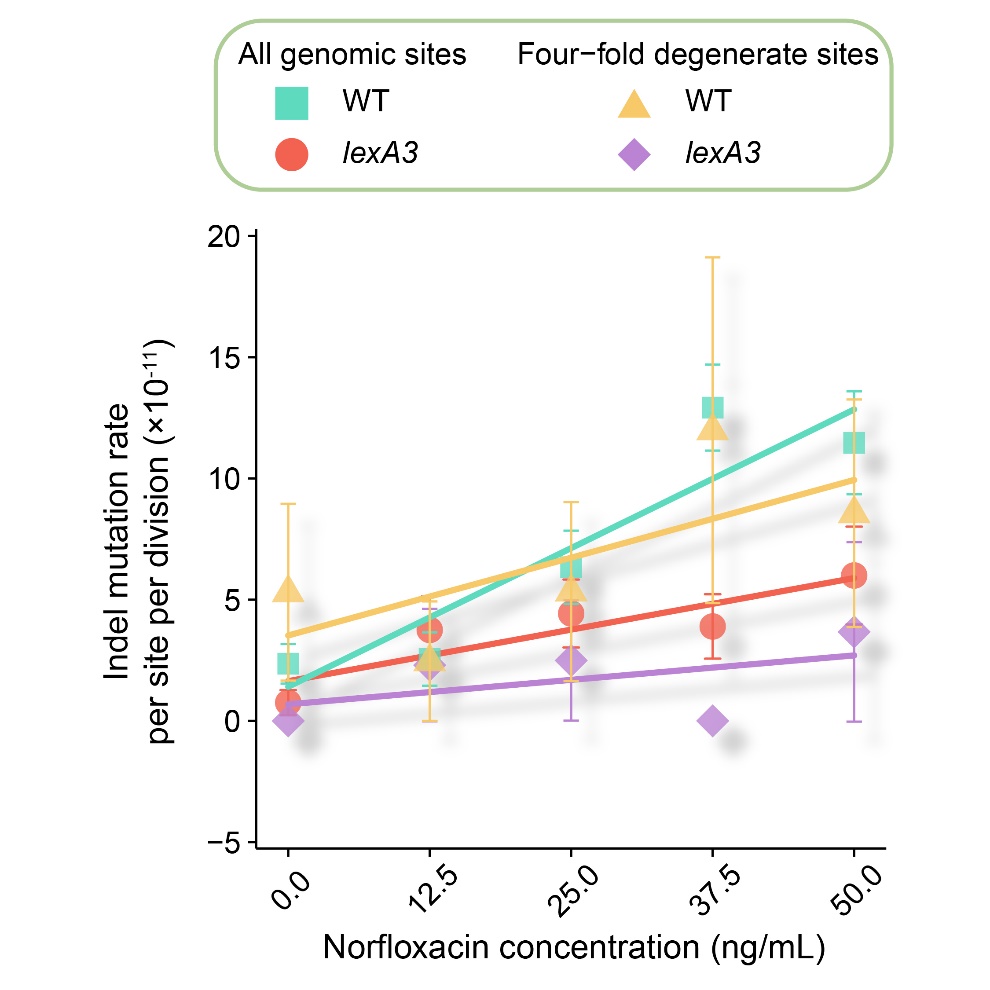
**

**Fig. S1** Mutation rates of small indels. Small-indel mutation rates of wild-type lines (WT) and SOS-uninducible lines (*lexA3*) treated with different doses of norfloxacin at all genomic sites and four-fold degenerate sites. Error bars are SEM.

**
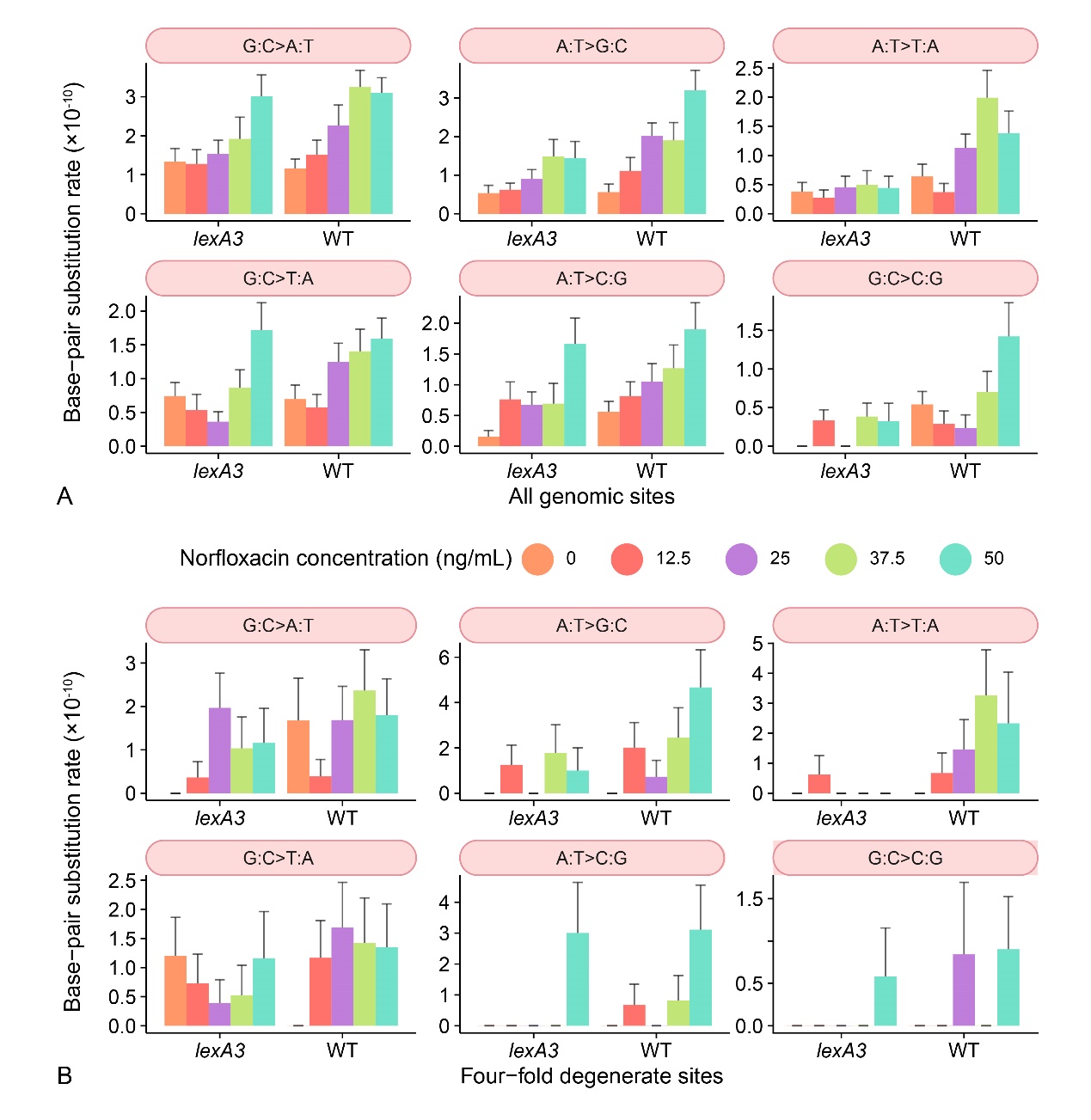
Fig. S2** Mutation spectra of the wild-type and SOS-uninducible (*lexA3*) lines treated with different norfloxacin doses at all genomic sites and four-fold degenerate sites. Bars denote SEM. **A** Mutation spectra of the wild-type and *lexA3* lines at all genomic sites. **B** Mutation spectra of wild-type and *lexA3* lines at four-fold degenerate sites.

**
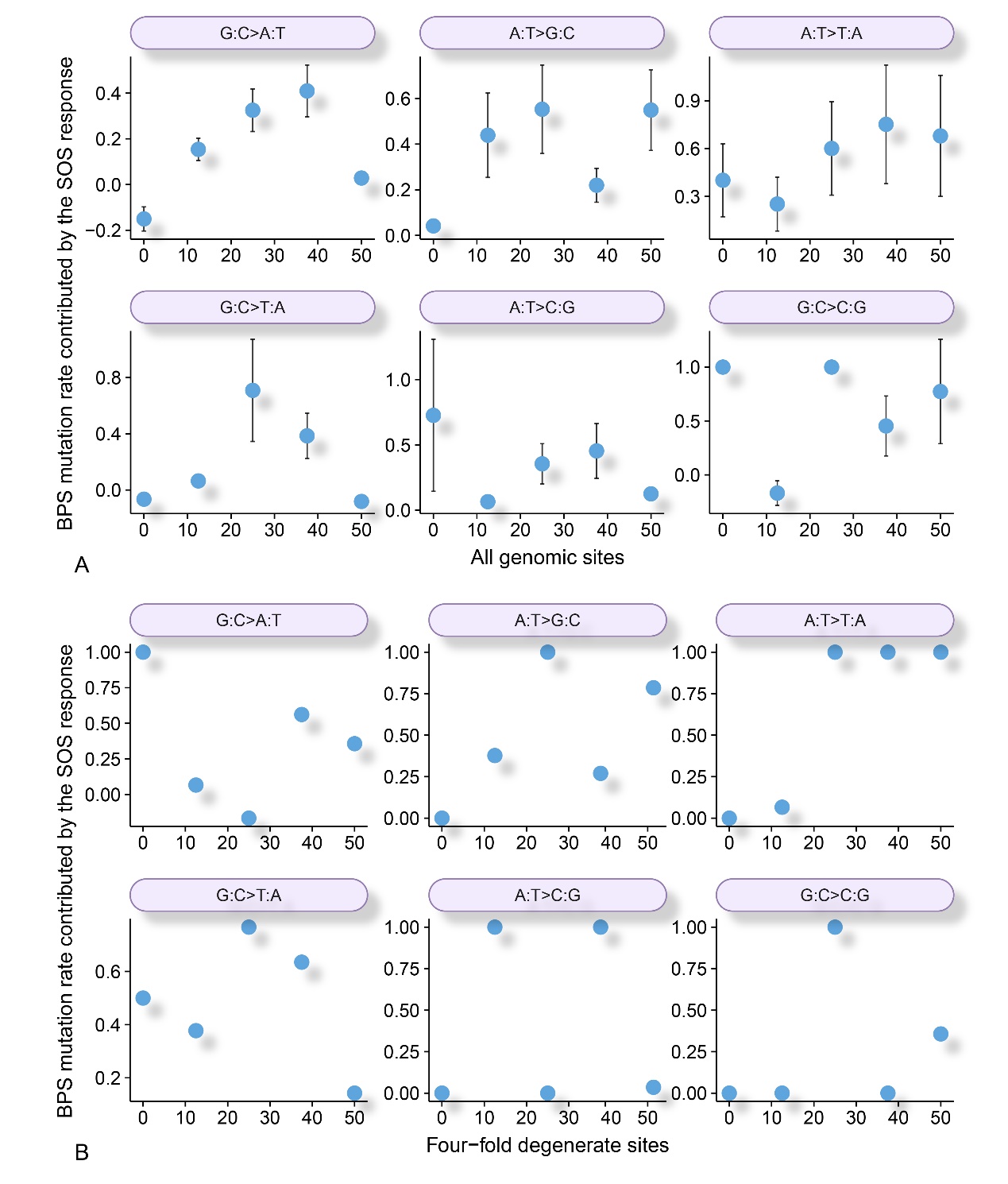
Fig. S3** Proportions of different types of BPS mutations elevated by the SOS response, at all genomic sites (**A**) and four-fold degenerate sites (**B**). X-axis represents norfloxacin concentrations (ng/mL). Error bars denote binomial-distribution SE.

**
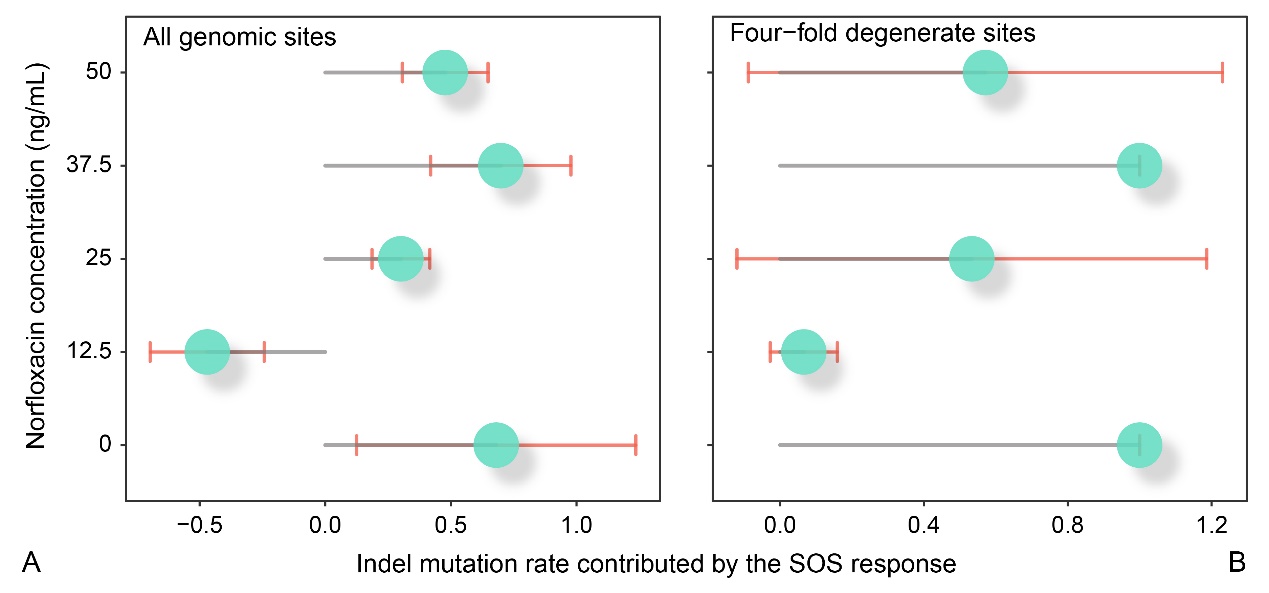
**

**Fig. S4** Proportion of small-indel mutations contributed by the SOS response under different norfloxacin doses at all genomic sites and four-fold degenerate sites. Error bars denote binomial-distribution SE. **A** Proportion of small-indel mutations contributed by the SOS response at all genomic sites, when treated with different doses of norfloxacin. **B** Proportion of small-indel mutations from the SOS response at four-fold degenerate sites, when treated with different doses of norfloxacin.

**
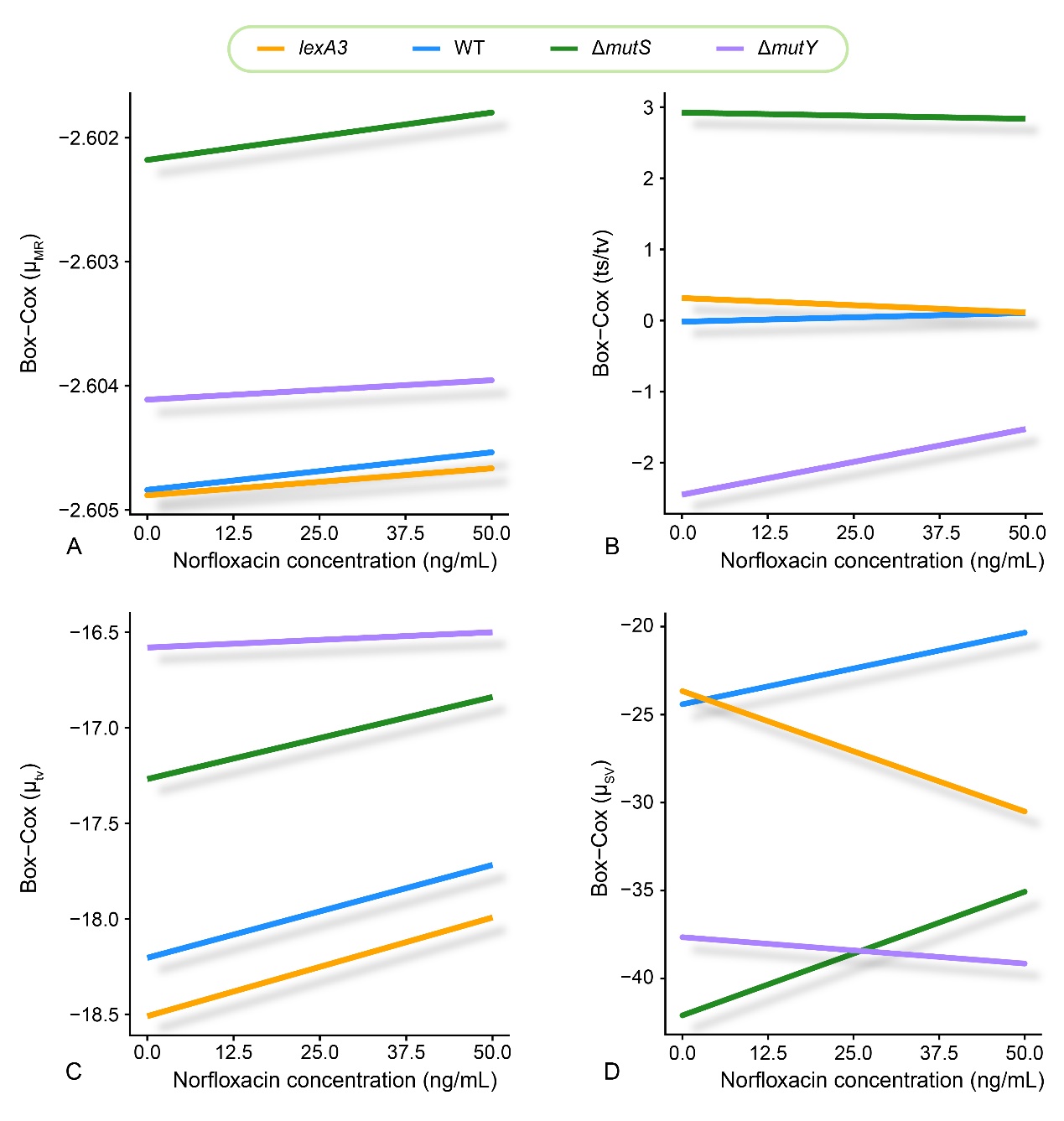
Fig. S5** The estimated relationships between the norfloxacin concentration vs. the BPS mutation rate, the ts/tv ratio, the transversion rate and the structural variation rate for the wild-type and the SOS-uninducible strains, revealed by Model 2. WT, the wild-type strain; *lexA3*, the SOS-uninducible strain; Δ*mutS*, the MMR-deficient strain; Δ*mutY*, the adenine DNA glycosylase-deficient strain. The position of the line represents the effect of the strain relative to the wild-type strain. The slopes reflect the contribution of these mechanisms to mutation rate upon norfloxacin change. **A** The estimated relationships between the norfloxacin concentration and the BPS mutation rate for the two strains, revealed by Model 2. There is an interaction term between norfloxacin concentration and strains. **B** The estimated relationships between norfloxacin concentration and the ts/tv ratio for the strains. **C** The estimated relationships between norfloxacin concentration and the transversion rate for the strains. **D** The estimated relationships between norfloxacin concentration and the SV rate for the strains.
