# Supplementary material for: Contribution of the SOS response and the DNA repair systems to norfloxacin induced mutations in E. coli
Source: Mar Life Sci Technol. 2023 Sep 21;5(4):538–50. doi: 10.1007/s42995-023-00185-y (PMC10689325; doi:10.1007/s42995-023-00185-y)
Supplement: Supplementary file 3 — Supplementary file3 (DOCX 44 KB) [file 42995_2023_185_MOESM3_ESM.docx]

**Contribution of the SOS response and the DNA repair systems to norfloxacin induced mutations in *E. coli***

**Tongtong Lin^#,1,2^, Jiao Pan^#,1^, Colin Gregory^#,3^, Yaohai Wang^1^, Clayton Tincher^3^, Caitlyn Rivera^3^, Michael Lynch^4^, Hongan Long^1,2^, Yu Zhang^*,1,5^**

1. Institute of Evolution and Marine Biodiversity, KLMME, Ocean University of China, Qingdao 266003, China
2. Laboratory for Marine Biology and Biotechnology, Laoshan Laboratory, Qingdao 266237, China
3. Department of Biology, Indiana University, Bloomington 47405, USA
4. Biodesign Center for Mechanisms of Evolution, Arizona State University, Tempe 85281, USA
5. School of Mathematics Science, Ocean University of China, Qingdao 266000, China

# These authors contributed equally to this work

* Corresponding author: Yu Zhang, zhangyu6929@ouc.edu.cn

**Supplementary File**

**Effects of the molecular mechanisms on the mutation spectrum**

The mutation spectrum can be depicted by the transitions to transversions ratio (ts/tv; we chose MA lines with both ts and tv being non-zero). The ts/tv data of all MA lines of the four strains were taken as input to calculate the influence of each mechanism on the ts/tv ratio without considering the effects of norfloxacin concentration (Table 4; Supplementary Table S13). The SOS response played a non-significant role on the ts/tv ratio, while the MMR and the oxidative damage repair were negatively and positively associated with the ts/tv ratio (*P* < 2 × 10^-16^), respectively. In Model 2, the norfloxacin concentration and interaction of norfloxacin concentration and the mechanisms were added to the linear model as explanatory variables to estimate the ts/tv ratio. The slope of the fitted model revealed that the SOS response and the MMR played a negative role in the increase of ts/tv ratio and increased with norfloxacin concentration (Table 5; Supplementary Fig. S5B). In addition, the higher slope of the Δ*mutY* strain demonstrated that oxidative damage repair significantly and positively correlated with the ts/tv ratio, but the trend decreased as the norfloxacin concentration increased. Moreover, the fitted lines of the SOS-uninducible and the wild-type strains crossed over at 50.0 ng/mL.

Such results reveal that the MMR and the oxidative damage repair might be the main determinants of the mutation spectrum and disentangle the power of the mechanisms in influencing the mutation spectrum upon norfloxacin treatment: given a norfloxacin concentration, the MMR and the oxidative damage repair are the dominating mechanisms in keeping the transition-dominant status and mutation biases in the A/T direction of the spectrum, although they work oppositely. As the norfloxacin concentration goes up, the ability of oxidative damage repair to reduce mutation biases in the A/T direction is weakened and so does its ability to elevate the ts/tv ratio, while the ability of the MMR to elevate mutation biases in the A/T direction and reduce the ts/tv ratio is both enhanced.

**Effects of the molecular mechanisms on the rate of transversions and structural variations**

Research has shown that the SOS response elevates transversions most conspicuously (Foster and Eisenstadt 1985; Wijker and Lafleur 1998), especially A:T🡪C:G transversions in the A:T base pairs. The overall transversion rates were not significantly different between the wild-type and the SOS-uninducible MA lines at all genomic sites and at any level of concentration (Supplementary Table S14). Only the A:T🡪T:A and A:T🡪C:G transversions at four-fold degenerate sites showed weak differences at some concentrations (Fig. 3B, D). To determine if the transversion-dominant spectra resulted from the SOS response, MMR or oxidative damage repair, we used a linear model with all the transversion rates of MA lines of wild-type, SOS-uninducible, Δ*mutS* and Δ*mutY* strains as observed data (Model 1). The fitted model showed that the SOS response was a significantly positive determinator (*P* = 0.0045; Table 5; Fig. 5B; Supplementary Table S15). By contrast, as expected, the MMR and oxidative damage repair systems had significantly negative effects on the transversion rate (*P* < 2.00 × 10^-16^; Table 5; Fig. 5B; Supplementary Table S15). We then estimated the effects of the three mechanisms on transversion rate under different norfloxacin concentrations by adding the variable N_C (norfloxacin concentration) to the model (Model 2). The effects of the MMR and the oxidative damage repair were negative on the transversion rate elevation upon norfloxacin treatment. However, the contribution of the SOS response to transversion rate increased with the norfloxacin concentration (Table 5; Supplementary Fig. S5C). This inferred that reactive oxygen species generation was not a major action mechanism for this fluoroquinolone, at least in the presence of norfloxacin, since ROS was known to oxidize nucleotides in the DNA strands or the cellular nucleotide pool, especially 8-oxo-guanines, and elevated transversions originated from oxidized nucleotides such as G:C🡪T:A and A:T🡪C:G transversions (Grollman and Moriya 1993; Michaels et al. 1992).

Besides BPSs and small indels, large-scale structural variations (SVs) may enable new adaptive phenotypes to arise and affect genome evolution differently from point mutations that only alter single DNA bases (Deatherage et al. 2015; Dunham et al. 2002; Gresham et al. 2008; Iguchi et al. 2006; Raeside et al. 2014). SVs include large deletions, gene duplications, mobile element insertions and other chromosomal rearrangements (Ho et al. 2020).

We thus explored the contribution of the above mutagenesis mechanisms to SVs, with or without norfloxacin treatment. Using breseq-0.35.1 (Barrick et al. 2014; Deatherage and Barrick 2014), we detected 99 SVs and 106 SVs in the SOS-uninducible and the wild-type MA lines treated with 0–50ng/mL norfloxacin, respectively (Supplementary Tables S16–S17), and most of them involved insertion sequence (IS) elements. Only one 120-bp deletion in the intergenic region of *iap* and *cas2* frequently occurred in the MA lines of both strains. Notably, the deletion occurred in the SOS-uninducible lines when the norfloxacin concentration was ≤ 25 ng/mL, while for the wild-type lines, such norfloxacin concentrations were ≥ 25 ng/mL. In the SOS-uninducible lines, the most frequent IS element insertion occurred between two genes that encode two ATP-dependent proteases: *clpX* and *lon* (Goldberg et al. 1994; Gottesman et al. 1993), after the norfloxacin concentration reached 25 ng/mL or above. IS2-mediated insertion in *bglH*, which is responsible for uptake and fermentation of *β*-glucosides and encodes a carbohydrate-specific outer membrane porin (Andersen et al. 1999), occurred at low to high norfloxacin concentrations in the SOS-uninducible MA lines. In addition, there were eight IS insertions that occurred at prophage coding regions in the SOS-uninducible lines and nine in the wild-type lines.

Next, we analyzed SVs of the Δ*mutS* and the Δ*mutY* MA lines treated with 0–50 ng/mL norfloxacin generated in one previous study (Long et al. 2016). We obtained 17 SVs from 60 Δ*mutS* MA lines and 45 SVs from 141 Δ*mutY* MA lines (Supplementary Tables S18–S19). 58.82% of the Δ*mutS* SVs were IS element-mediated insertions and the remaining were large-scale deletions. In the Δ*mutS* MA line, the largest deletion was 35,590 bp, observed in groups SC and SE. For the Δ*mutY* MA lines, most of the SVs were IS element-mediated insertions (IS5 and IS1), only 22.22% of them were large-scale deletions containing 12 genes, and the length of the most common deletion was 11,471bp. The deletion was detected when the norfloxacin concentration reached 25 ng/mL or above.

We also used the SV rate of all MA lines of wild-type, SOS-uninducible, Δ*mutS* and Δ*mutY* strains to fit Model 1. The results reveal that all three mechanisms (SOS response, MMR and oxidative damage repair) are positively correlated with the SV rates, but only the MMR and the oxidative damage repair show significance (Table 4; Supplementary Table S20; Fig. 5C). Then we added the variable of norfloxacin concentration to the mode (Model 2). The strength of the MMR decreases with the norfloxacin concentration, while those of the SOS response and the oxidative damage repair show the opposite pattern as the norfloxacin concentration increases (Table 5; Supplementary Fig. S5D).

**References**

Andersen C, Rak B, Benz R (1999) The gene *bglH* present in the *bgl* operon of *Escherichia coli*, responsible for uptake and fermentation of β-glucosides encodes for a carbohydrate-specific outer membrane porin. Mol Microbiol 31:499-510

Barrick JE, Colburn G, Deatherage DE, Traverse CC, Strand MD, Borges JJ, Knoester DB, Reba A, Meyer AG (2014) Identifying structural variation in haploid microbial genomes from short-read resequencing data using breseq. BMC Genomics 15:1-17

Deatherage DE, Barrick JE (2014) Identification of mutations in laboratory-evolved microbes from next-generation sequencing data using breseq. In: Sun L, Shou W (eds) Engineering and analyzing multicellular systems. Methods in molecular biology, vol 1151. Humana Press, New York, pp 165-188.

Deatherage DE, Traverse CC, Wolf LN, Barrick JE (2015) Detecting rare structural variation in evolving microbial populations from new sequence junctions using breseq. Front Genet 5:468

Dunham MJ, Badrane H, Ferea T, Adams J, Brown PO, Rosenzweig F, Botstein D (2002) Characteristic genome rearrangements in experimental evolution of *Saccharomyces cerevisiae*. Proc Natl Acad Sci 99:16144-16149

Foster PL, Eisenstadt E (1985) Induction of transversion mutations in *Escherichia coli* by *N*-methyl-*N'*-nitro-*N*-nitrosoguanidine is SOS dependent. J Bacteriol 163:213-220

Goldberg AL, Moerschell RP, Hachung C, Maurizi MR (1994) ATP-dependent protease La (Lon) from *Escherichia coli*. Methods Enzymol 244:350-375

Gottesman S, Clark WP, de Crecy-Lagard V, Maurizi MR (1993) ClpX, an alternative subunit for the ATP-dependent Clp protease of *Escherichia coli.* Sequence and in vivo activities. J Biol Chem 268:22618-22626

Gresham D, Desai MM, Tucker CM, Jenq HT, Pai DA, Ward A, DeSevo CG, Botstein D, Dunham M (2008) The repertoire and dynamics of evolutionary adaptations to controlled nutrient-limited environments in yeast. PLoS Genet 4:e1000303

Grollman AP, Moriya M (1993) Mutagenesis by 8-oxoguanine: an enemy within. Trends Genet 9:246-249

Ho SS, Urban AE, Mills RE (2020) Structural variation in the sequencing era. Nat Rev Genet 21:171-189

Iguchi A, Iyoda S, Terajima J, Watanabe H, Osawa R (2006) Spontaneous recombination between homologous prophage regions causes large-scale inversions within the *Escherichia coli* O157: H7 chromosome. Gene 372:199-207

Long H, Miller SF, Strauss C, Zhao C, Cheng L, Ye Z, Griffin K, Te R, Lee H, Chen C-C, Lynch M (2016) Antibiotic treatment enhances the genome-wide mutation rate of target cells. Proc Natl Acad Sci 113:E2498-E2505

Michaels ML, Cruz C, Grollman AP, Miller JH (1992) Evidence that MutY and MutM combine to prevent mutations by an oxidatively damaged form of guanine in DNA. Proc Natl Acad Sci 89:7022-7025

Raeside C, Gaffé J, Deatherage DE, Tenaillon O, Briska AM, Ptashkin RN, Cruveiller S, Médigue C, Lenski RE, Barrick JE (2014) Large chromosomal rearrangements during a long-term evolution experiment with *Escherichia coli*. mBio 5:e01377-14

Wijker CA, Lafleur MVM (1998) Influence of the UV-activated SOS response on the *γ*-radiation-induced mutation spectrum in the *lacI* gene. Mutat Res 408:195-201
